# Discovery and Further Studies on Giant Viruses at the IHU Mediterranee Infection That Modified the Perception of the Virosphere

**DOI:** 10.3390/v11040312

**Published:** 2019-03-30

**Authors:** Clara Rolland, Julien Andreani, Amina Cherif Louazani, Sarah Aherfi, Rania Francis, Rodrigo Rodrigues, Ludmila Santos Silva, Dehia Sahmi, Said Mougari, Nisrine Chelkha, Meriem Bekliz, Lorena Silva, Felipe Assis, Fábio Dornas, Jacques Yaacoub Bou Khalil, Isabelle Pagnier, Christelle Desnues, Anthony Levasseur, Philippe Colson, Jônatas Abrahão, Bernard La Scola

**Affiliations:** 1 MEPHI, APHM, IRD 198, Aix Marseille Univ, Department of Medicine, IHU-Méditerranée Infection, 13005 Marseille, France; rolland.clara@sfr.fr (C.R.); miaguiabidou@gmail.com (J.A.); cheriflamina@gmail.com (A.C.L.); aherfi.s@gmail.com (S.A.); raniagfrancis@gmail.com (R.F.); rodriguesral07@gmail.com (R.R.); ludmilakaren@gmail.com (L.S.S.); medecine.to@hotmail.com (D.S.); saidmougari@ymail.com (S.M.); nisrine.chelkha@hotmail.fr (N.C.); meriembekliz@hotmail.com (M.B.); lorena.farmacia@yahoo.com.br (L.S.); felipelopesassis@gmail.com (F.A.); fabiopiod154@gmail.com (F.D.); isabelle.pagnier@univ-amu.fr (I.P.); christelle.desnues@univ-amu.fr (C.D.); anthony.levasseur@univ-amu.fr (A.L.); philippe.colson@univ-amu.fr (P.C.); jonatas.abrahao@gmail.com (J.A.); 2 Laboratório de Vírus, Instituto de Ciêncas Biológicas, Departamento de Microbiologia, Universidade Federal de Minas Gerais, 31270-901 Belo Horizonte, Brazil; 3 IHU IHU-Méditerranée Infection, 13005 Marseille, France; boukhaliljacques@gmail.com

**Keywords:** giant virus, amoeba, *Mimivirus*, virosphere

## Abstract

The history of giant viruses began in 2003 with the identification of *Acanthamoeba polyphaga mimivirus.* Since then, giant viruses of amoeba enlightened an unknown part of the viral world, and every discovery and characterization of a new giant virus modifies our perception of the virosphere. This notably includes their exceptional virion sizes from 200 nm to 2 µm and their genomic complexity with length, number of genes, and functions such as translational components never seen before. Even more surprising, *Mimivirus* possesses a unique mobilome composed of virophages, transpovirons, and a defense system against virophages named *Mimivirus* virophage resistance element (MIMIVIRE). From the discovery and isolation of new giant viruses to their possible roles in humans, this review shows the active contribution of the University Hospital Institute (IHU) Mediterranee Infection to the growing knowledge of the giant viruses’ field.

## 1. Introduction

For this 10th-year anniversary special issue of the journal *Viruses,* members or former members (mostly former PhD) of our team working at the University Hospital Institute (IHU) Mediterranee Infection prepared this review that presents the last 15 years of our work on giant viruses, from the discovery and description of *Mimivirus* to our latest studies, some under review and others not yet completed.

## 2. The History of Discovery of Giant Viruses and Their Genetic Mobile Elements

**Figure 1:**
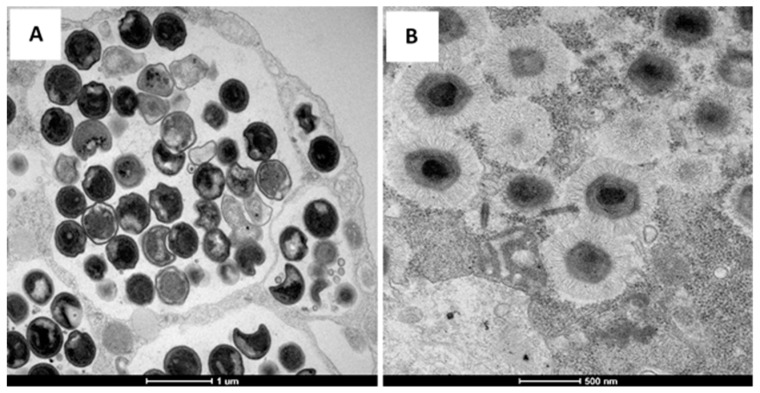
Transmission electron microscopy of the *Chlamydia* bacterium (**A**) and *Mimivirus* (**B**).

Our former laboratory, URMITE, a research unit on emerging infectious and tropical diseases, reference center for *Rickettsia* and rickettsial diseases, was traditionally expert in the isolation and study of fastidious and intracellular microorganisms. In the 1990s, we were the first French hospital laboratory to routinely identify rare bacteria using 16S ribosomal RNA (rRNA) gene sequencing [1]. In the same period, we began using amoebas as cell supports for the isolation of intracellular bacteria, mostly *Legionella,* but also as a means to isolate, from human and environmental samples, potential new agents responsible for pneumonia that would not grow on axenic culture media [2,3,4,5,6,7,8]. Amoeba co-culture and identification based on the 16S rRNA sequence led us to collaborate with Dr. Tim Rowbotham, the scientist who described the isolation of *Legionella* using amoeba co-culture [9]; in 1995, a post-doctoral student from his laboratory, Richard Birtles, came to Marseille to identify his collection of amoeba-associated bacterial isolates. Among the bacteria of this collection, most being identified as *Legionella-*like amoebal pathogens by 16S rRNA gene sequencing [10], there was a small Gram-positive coccoid bacterium provisionally named “Bradford coccus”. This bacterium was later resistant to molecular analysis and we decided that examination of the ultrastructure of Bradford coccus using electron microscopy might provide us with a solution to our technical problems, i.e., a particular cell wall that would protect DNA from extraction. During examination, we observed unexpected regular icosahedral bodies that were typical of a virus but with the size of a small bacterium (Figure 1). We observed for the first time a particle belonging to a new group of viruses, giant viruses, with its first member *Acanthamoeba polyphaga mimivirus* (APMV) [11]. Its size of ~600 nm with its surrounding fibrils, the size of its genome of 1.2 Mbp, and its coding capacity including genes/functions never observed in a virus let us imagine that this discovery could deeply modify the perception we had of the virosphere [12]. The next discoveries we made (as well as others) on the subject gradually confirmed this first impression (Table 1). However, at the stage of this manuscript, it should be noted that *Mimivirus*, seen as a unique circus freak in 2003, is now, 15 years later, one of the most diverse microorganisms, at least in sea water [13].

We firstly started to look specifically for giant viruses using amoeba co-culture, and we could identify three different lineages in the *Mimivirus* family: lineage of *Mimivirus stricto sensu* (lineage A), lineage of *Moumouvirus* (lineage B), and lineage of CE11 (lineage C) [14]. This lineage C was later named the *Megavirus* lineage [48,49]. During the search for giant virus isolates, we succeeded in isolating a new giant virus, *Marseillevirus* [15] and a curious *Mimivirus* with slow multiplication and particles seeming larger than the original APMV we named *Mamavirus*. Electron microscopy observations showed that it appeared larger due to particles with abnormal layers of particle wall, and a small virus of 50 nm in diameter was observed as developing within its viral factory [34]. This small virus we named Sputnik was in fact a virus that infected the APMV virus factory, and we further defined it as a virophage by analogy with bacteriophages that are virus-infecting bacteria. Subsequently, we were able to identify that the genome of a virophage could be integrated into the genome of a giant virus and we named it a provirophage [35]. In the same study, while searching for other mobile elements in giant viruses, we also detected transposons we named transpovirons. In later works, we searched for new strains of virophages using reporter giant viruses allowing the isolation of Sputnik 3 [25] and Guarani virophage [37]. One of the new virophages named Zamilon had the originality to infect lineage B and C of *Mimivirus* but not lineage A. We imagined that this resistance could be due to a CRISPR/Cas system, a system allowing bacteria to resist phages [50]. However, analysis of the APMV genome did not reveal any significant homology with enzymes of a CRISPR/Cas system. As a CRISPR/Cas system consists of enzymes but also of repeats and integration of the targeted bacteriophage sequence, we then searched for repeats and virophage sequences in the APMV genome. The genome of Zamilon was fragmented into short fragments of 40 nucleotides using a sliding window of 10 nucleotides (nts), and all fragments were compared using BLAST software against the respective APMV genomes. We were surprised to find, in the lineage A *Mimiviruses*, four repeats of 15 nts corresponding to Zamilon. Observation in the vicinity of these repeats of nuclease and helicase genes allowed us to suspect that this operon that we named MIMIVIRE for *Mimivirus* resistance element could be the defense mechanism of lineage A *Mimivirus* againt the Zamilon virophage. Silencing of this operon confirmed this hypothesis [38]. We were recently able to isolate a *Mimivirus* isolate of lineage A with a modified MIMIVIRE operon that is susceptible to Zamilon; via transient knock-out (KO) of the MIMIVIRE operon, we could restore susceptibility of APMV to the Zamilon virophage [31].

**Figure 2:**
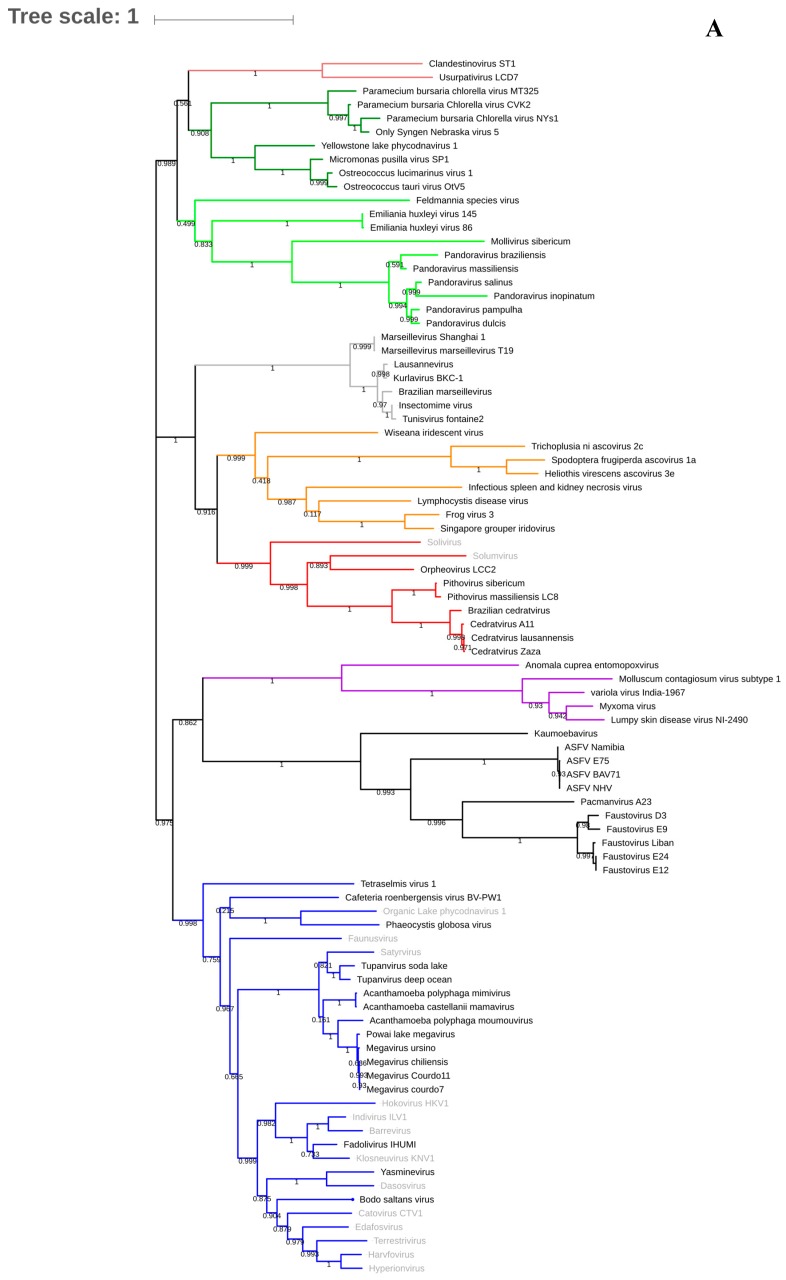

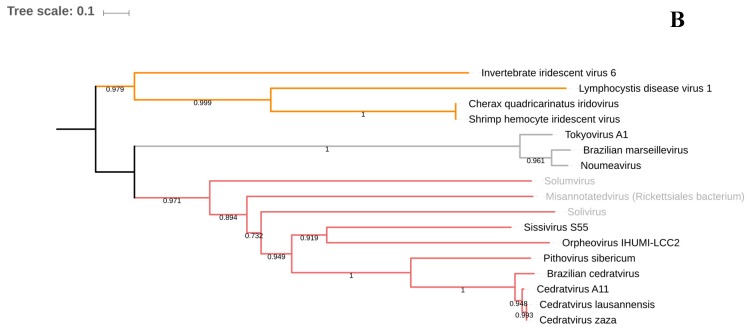
Phylogenetic trees based on DNA polymerase B of giant viruses (**A**) and on *VETF* gene to illustrate the clustering of *Pithoviridae* and *Sissivirus* (**B**). The viruses described only in metagenomics are labeled in gray. The analysis was performed using Muscle and FastTree, applying the maximum-likelihood method with 1000 bootstrap replicates.

If metagenomics, especially with last-generation high-throughput sequencing, recently demonstrated an extraordinary potential to discover new families of giant viruses [51,52], we continue to believe that virus isolation attempts are not an outdated strategy. Following the discovery of *Mimivirus*, our laboratory, with the successful collaboration of the Virology Laboratory of the Federal University de Minas Gerais, continued its efforts to isolate new giant/protozoa-associated viruses. This search followed three major axes that we developed: building high-throughput isolation techniques, diversifying the amoeba supports, and searching in original biotopes. This was efficient for the discovery of new viruses like *Faustovirus*, *Kaumoebavirus*, *Pacmanvirus*, *Orpheovirus*, *Cedratvirus*, and *Tupanvirus* [18,19,20,21,22,23]. We also managed to isolate members of families described by our colleagues such as *Pandoraviruses* or *Pithoviruses* [16,17,53,54]. Moreover, we are currently analyzing new viruses we recently isolated, all of them representing new families, including *Yasminevirus* and *Fadolivirus*, the first isolated members of the putative family of *Klosneuvirinae* [51], as well as viruses never detected to date using metagenomics such as *Clandestinovirus* and *Usurpativirus* (Unpublished under characterization) (Figure 2).

## 3. History and Revolution of the Giant Viruses’ Isolation Process through the Years

Many studies confirmed giant virus ubiquity and diversity in the environment and in humans [55,56,57,58]. Therefore, isolating the viral particle remains crucial in order to access its genetic content and perform further analysis allowing a better understanding of this new emerging world. Their natural host remains unknown, and co-culture on amoeba remains the only key engine for isolating these viruses. This strategy, although it allowed the isolation of the first *Acanthamoeba polyphaga mimivirus* [11], remained inefficient for many years before it could isolate other giant viruses, suggesting the need to improve, standardize, and automate the co-culture to a level that would allow more isolates to be produced. In this review, we summarize the main improvements brought to the giant virus isolation strategies in our lab over the years along with a brief descriptive timeline of the main modifications that marked the amoebal co-culture and giant virus isolation (Table 2).

Two cases are discussed regarding this issue; one is related to the samples and the cell hosts, and the other is related to the tools used to reduce the time of co-culture and enhance the isolation rate.

The “amoebal enrichment method” in shell vials was first implemented by T.J. Rowbotham to isolate *Legionella* species [9]. This strategy allowed the isolation of *Acanthamoeba polyphaga mimivirus* in 2003 [11] and was adopted for future giant virus isolation processes. However, this technique remained fastidious for many years due to the lack of expertise in the field. Many attempts were made to improve the isolation strategy starting with the diversification of the samples where giant viruses were reportedly isolated from various environments such as permafrosts, lakes, sewage, soil, water, human samples, and many others [19,39,45,53,59]. Other improvements involved the pre-treatment of the samples prior to co-culture by means of filtration, precipitation, pre-enrichment, and antibiotic and antifungal treatments. Many isolates were retrieved from treated samples notably *Pithovirus sibericum* [53], *Mollivirus sibericum* [60], *Pandoravirus salinus* and *dulcis* [54], *Megavirus chilensis* [48], *Samba virus* [61], and others. These improvements are fairly detailed in a previous review written by Bou Khalil et al. [62]. Many studies later proved that enlarging the panel of amoeba used for co-culture allowed the recovery of different isolates, whereby a single giant virus can be positive for a certain amoeba but negative for another [63]. For many years, we used *Acanthamoeba polyphaga* as a cell support for the isolation procedure, but we never managed to isolate new viruses other than *Mimivirus* or *Marseillevirus*, whereas other teams using *Acanthamoeba castellanii* successively isolated *Pandoravirus*, *Mollivirus*, and *Pithovirus*. For this, and after using diverse cell hosts, we managed to isolate many new strains of giant viruses [21,22,23]. This concept also allowed the isolation of the first *Faustovirus* on *Vermamoeba vermiformis* [18] and recently the isolation of *Orpheovirus* IHU-MI [21].

From a different perspective, Boughalmi et al. introduced in 2012 a novel way of detecting amoebal lysis with the naked eye using co-culture on agar plates [24]. This technique allowed the isolation of new representatives of the *Mimiviridae* and *Marseilleviridae* families. However, it was still fastidious and limited to the use of protozoa growing on agar plates, which increased the risk of cross-contamination between samples. In 2013, the co-culture on amoeba was adapted to the use of microplates. Antibiotics and antifungal were also used in order to eliminate contaminants coming from the sample [64]. However, until that time, the isolation strategy was still fastidious, highly time-consuming, and operator-dependent, and it presented a low yield compared to the ubiquity and diversity of giant viruses in the environment reported by metagenomic studies [55,56,57,58]. This suggested the need for new automated tools to be used for the rapid isolation and identification of giant viruses. Therefore, in 2016, Bou Khalil et al. introduced flow cytometry in liquid medium to the isolation process. This tool allowed the automated detection of cell burst, the presumptive identification of giant viruses based on their DNA content [26], and the sorting of viral mixtures [27]. This technique also allowed the use of highly motile protozoa as cell hosts for the co-culture. In addition, the enrichment steps were optimized and coupled with new targeted antibiotic and antifungal mixtures, which dramatically reduced the time for each step. However, flow cytometry is a blind technique and presents a high risk of contamination. To overcome these limitations, we recently developed a new isolation strategy based on an automated high-content screening of co-culture and rapid scanning electron microscopy identification [65]). These new tools will expand the research spectrum in terms of varying samples and hosts for a more efficient hunt of giant viruses. To summarize, the co-culture process witnessed many improvements and modifications through the years, where some were fruitful and others remained less useful. However, we could learn much from this chronological overview to summarize a reliable strategy that could be adapted and standardized by all trackers of giant viruses by applying the same protocols of sample preparation and using a large panel of potential protozoa capable of harvesting or producing giant viruses. Automation of detection was one of the biggest achievements realized in our lab that revolutionized the giant virus isolation step and yielded hundreds of new strains. This process will continue to be developed and improved in our lab in order to get artificial intelligence software for correlative microscopy capable of easily identifying new isolates.

## 4. *Mimiviridae*

Following the outstanding discovery and characterization of *Acanthamoeba polyphaga mimivirus* (APMV), a new family, named *Mimiviridae,* was created to encompass this virus and other new members that also present similar morphological and genetic features [11,12,66]. Currently, this family includes two genera recognized by the International Committee of Taxonomy of Viruses (ICTV): *Mimivirus* and *Cafeteriavirus.* The IHU Mediterranee Infection team isolated hundreds of mimiviruses from a plethora of environmental and clinical samples, which increased knowledge about the exceptional diversity of this group of viruses [66]. Currently available data suggest the division of mimiviruses into at least three lineages (A, B, and C) [40,48,61,63,66,67,68,69]. The genus *Cafeteriavirus* comprises a single species, *Cafeteria roenbergensis virus* (CRoV), in which particles infect marine flagellates [70]. Both mimiviruses and CRoV were described to be parasited by virophages, and complex relationships were described, involving protists, giant viruses, an exclusive mobilome, and virophages [35,38,71].

Recently, phylogenomic analyses of viruses, previously considered as *Phycodnaviridae,* showed that they are actually closer to other mimiviruses than phycodnaviruses [72,73]. The description of this group of viruses, now denominated as extended mimiviruses, represented the beginning of a substantial expansion of *Mimiviridae* that was followed by other important discoveries. In 2017, metagenomic data identified the genome of four different *Mimiviridae*, with an expanded complement of translation factors, named *Klosneuvirus* [51]. In 2018, a member of *Klosneuvirus* group, named *Bodo saltans* virus, was isolated for the first time from a kinetoplastid protozoan [74]. We recently isolated two members of this *Klosneuvirinae* subfamily that are currently under characterization, *Fadolivirus* and *Yasminevirus*.

**Figure 3:**
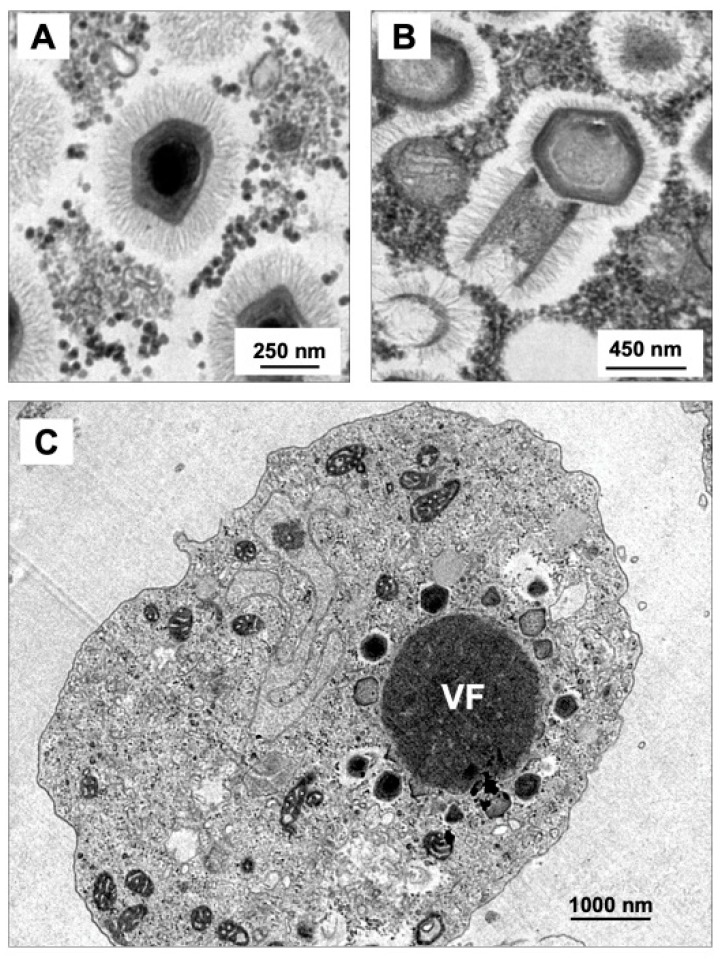
Transmission electron microscopy of *Mimivirus* (**A**) and *Tupanvirus* particles (**B**). (**C**) *Tupanvirus* viral factory, occupying a large portion of *Acanthamoeba castellanii* cytoplasm. VF: viral factory.

A continuous search for giant virus relatives in extreme environments led the IHU/UFMG teams to the discovery of *Tupanviruses*. Isolated from samples collected in soda lake and oceanic sediments in Brazil, these viruses present similar characteristics with mimiviruses of amoebae, but also unique and distinctive features never observed before, including a tailed particle and the most complete set of translation-related genes (Figure 3) [19,75]. A new genus, named “*Tupanvirus*”, was recently proposed to include the species “*Tupanvirus soda lake*” and “*Tupanvirus deep ocean*” [76]. *Tupanvirus* presents a broad host-range, infecting not only *Acanthamoeba* genus, as described with other amoebal mimiviruses [77,78,79,80,81], but also *Vermoameba vermiformis*, *Dyctiostelium discodeum*, and *Willeartia magna*. A toxic profile induced by *Tupanvirus* was described, related to the shutdown of host and non-host rRNA [19,75]. Controversial and exciting topics regarding *Mimiviridae* were raised in recent years, including their potential pathogenicity to humans and their position in the tree/rhizome of life [80,81,82,83,84,85,86,87,88]. *Mimivirus* studies represent an open field for remarkable discoveries and fundamental debates regarding the origins of nucleocytoplasmic large DNA viruses (NCLDVs) and cellular organisms.

## 5. *Marseilleviridae*

The *Marseilleviridae* family was the second family of giant viruses to be described [89]. *Marseillevirus marseillevirus* (MSRV), the prototype virus, was discovered in 2007 in our laboratory [15]. Its particle and genome are smaller than those of mimiviruses but still giant for viruses [90]. In 2018, 15 *Marseillevirus* isolates were described including six at the IHU Méditerranée Infection (Table 3) [14,15,32,42,91,92,93,94,95,96,97,98,99,100]. These viruses have diverse geographical origin (nine countries, five continents) and were retrieved from various environmental samples including waters, soils, insects, and mussels. They were isolated by co-culture with *Acanthamoeba castellanii* or *Acanthamoeba polyphaga*, and they were classified into five lineages.

The analysis of their replication cycle showed different pathways to enter into amoebae including phagocytosis of vesicles containing hundreds of particles, and endocytosis of single particles [101]. The replicative cycle is completed 12 h post infection (p.i.) with an eclipse phase at 2 h p.i., before the appearance of a large viral factory in the host cytoplasm at 4 h p.i. where virion morphogenesis occurs, and a complete lysis of amoebae at 12 h p.i. with the release of the new viral progeny [101]. Marseilleviruses have an icosahedral capsid of about 250 nm, with small fibrils of 12 nm described for MSRV, enclosing circular double-stranded DNA genomes ranging in size from 346 to 386 kilobase pairs (kbp), predicted to encode between 386 and 491 proteins. Gene repertoires encompass the giant virus core genes along with genes unique to giant viruses including some involved in transcription and a few genes related to the translation process, paralogous genes, and large fractions of genes with no homolog outside the viral family or known function [90]. In addition, the genome of these viruses is characterized by a substantial level of mosaicism with sequences of different putative origins, including eukaryotic, bacterial, archaeal, and viral [64]. Transcriptome analysis of MSRV confirmed the existence of predicted genes and revealed a temporal expression pattern, but no correlation with conserved AT-rich putative promoter motifs present in single or multiple copies [102] over more than 50% of the genes [103].

Moreover, marseilleviruses were detected in several samples collected from both symptomatic [45,46,47,101] and asymptomatic humans [42,43,104], being isolated once from a healthy man (see chapter on giant viruses in humans). Also, an up to 30-day-long persistence of MSRV in rats and mice was observed following intraperitoneal, intravenous, or airway inoculation [105]. The possible pathogenic nature of these viruses is still under investigation.

## 6. *Faustoviruses* and *Asfarvirus*-Related Giant Viruses of Amoeba

**Figure 4:**
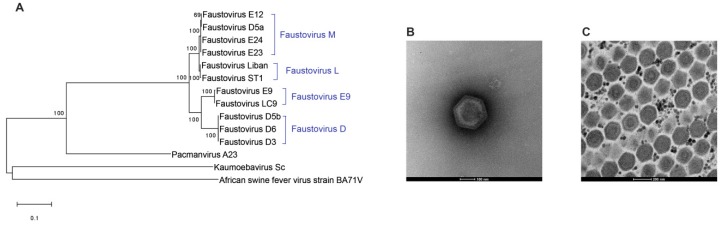
(**A**) DNA-directed RNA polymerase beta subunit-based tree illustrating the clustering of *Faustovirus* isolates in four lineages, *Kaumoebavirus*, and *Pacmanvirus*. The maximum-likelihood method and Jones–Taylor–Thornton model for amino-acid substitution were used with 1000 bootstrap replicates. (**B**) Negative staining of *Faustovirus* ST1 purified suspension showing an icosahedral particle of 200 nm. (**C**) Electron microscopy of the honeycomb structure of *Pacmanvirus* A23 viral factory in *Acanthamoeba castellanii*.

*Faustoviruses* were the first described giant viruses to infect an amoeba from a genus distinct from the commonly used *Acanthamoeba* species [18]. Considering the reported high abundance of *Vermamoeba vermiformis* in diverse environmental and human samples, (hospital water networks [106,107], drinking water [108], human stool samples [109], and contact lenses of keratitis patients [110,111]), its role as a reservoir for pathogenic agents was hypothesized. Our team, thus, incorporated this amoeba to the panel of cell supports in the high-throughput co-culture protocols allowing the successful isolation of *Faustovirus* E12, the prototype strain, from a sewage sample [18]. To date, we described and sequenced the genomes of 11 *Faustovirus* isolates classified in four lineages (Figure 4A). All isolates were recovered from sewage samples collected in different geographical locations (France, Senegal, Lebanon), but *Faustovirus*-like sequences were otherwise identified in diverse biomes including *Culicoides* guts, cattle sera, rodent organs, and human healthy and febrile patient sera [18,33,112,113].

*Faustovirus* E12 replication cycle in *V. vermiformis* lasts 18 to 20 h after individual viral particles’ phagocytosis by the amoeba. The eclipse phase occurs from 4 to 6 h p.i. along with a reorganization of the host nucleus. From 8 to 10 h p.i., a donut-shaped virus factory appears with newly formed viral particles, released through cell lysis in the last step of the cycle [18]. Viruses of this group form icosahedral virions of 200–240 nm in diameter containing a double-stranded DNA genome of 456 to 491 kilobase pairs (kbp) predicted to encode for 477 to 519 genes. A large proportion of these genes (~70%) encode for proteins whose functions are not yet identified due to the absence of known homologs. The comparative genomic study of nine *Faustovirus* isolates showed an open pan-genome of over 1000 genes and a stable core-genome of around 207 genes, 74% of which have best matches with members of the *Asfarviridae* family [114].

Among giant viruses, *Faustoviruses* are most closely related to the *African swine fever virus* (ASFV), a tick-borne virus responsible of highly epidemic hemorrhagic fever in domestic pigs. This is most clearly demonstrated when DNA polymerases are used for phylogenetic studies [84]. Interestingly, while these two viruses share a similar gene expression pattern and the highest proportion of homologous proteins, faustoviruses stand out with the size of their genomes, the complexity of their gene structure, and the double-protein shell that forms their capsid [115]. In *Faustovirus* E12, the gene coding for the major capsid protein (MCP) forming the external protein layer of the capsid is 17 kbp long and comprises 13 exons separated by large group I and spliceosomal-like introns defined by non-canonical splice sites [116]. Homology searches of the *MCP* gene in other faustoviruses showed the presence of six different splicing profiles that correlate with the described lineages (Unpublished under characterization).

*Kaumoebavirus* strain Sc was the second giant virus of amoeba isolated on *Vermamoeba vermiformis* [20] (Figure 4B). Although phylogenetically distant from faustoviruses and with a unique genome topology, *Kaumoebavirus* shares several characteristics with faustoviruses: (i) a comparable morphology with 250-nm large icosahedral particles protected by a double-layered capsid, (ii) replication cycles of similar lengths in their common amoebal host, and (iii) the spliced structure of the *MCP* gene, predicted to be 5 kbp long in *Kaumoebavirus*. Moreover, a recent study showed the presence of an AT-rich motif similar to a known promotor of ASFV in the intergenic regions of *Faustoviruses* and *Kaumoebavirus* suggesting a potential shared gene expression regulation mechanism in these viruses [117].

In the *Asfarvirus*-related giant viruses of amoeba, *Pacmanvirus* was isolated on *Acanthamoeba castellanii* (Figure 4C). This virus induces amoebal lysis in 6 to 8 h and produces icosahedral particles of the same size range as *Faustoviruses* and *Kaumoebavirus* (250 nm), harboring a genome of intermediate length (395 kbp). *Pacmanvirus* shares 31 genes with *Faustovirus*, *Asfarvirus*, and *Kaumeobavirus*, but is the only virus in this group that encodes for a transfer RNA (tRNA; isoleucine-tRNA) [23].

## 7. *Pithoviridae* and Related Viruses

**Figure 5:**
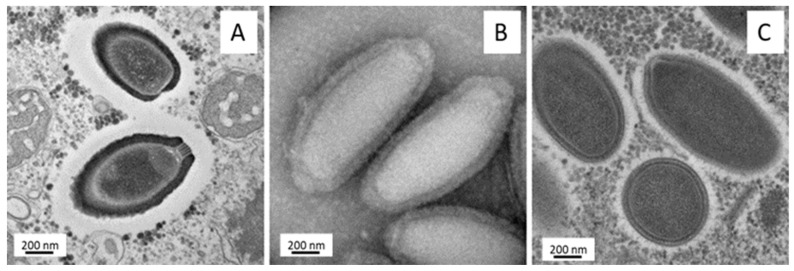
Transmission electron microscopy of *Pithovirus massiliensis* (**A**), *Cedratvirus* A11 (**B**), and *Orpheovirus* particles (**C**).

The first member of this group of giant viruses was isolated from a >30,000-year-old Siberian permafrost sample and was named *Pithovirus sibericum* [53]. This virus has the largest elipsoid viral particle to date, with a mean size of 1.5 µm in length, exhibiting a single cork in one extremity, from where the genome is released [53] (Figure 5A). Despite this first observation, structural analysis permitted discovering discrete forms for *Pithovirus sibericum* with two corks [118]. A contemporary virus was isolated in 2016 from French sewage samples, exhibiting similar virion structure and a genetic conservation compared to its prehistoric counterpart [17]. Their genome is a circular double-stranded DNA molecule of 610–683 kb coding for up to 520 genes, with a large fraction (~21%) corresponding to multiple palindromic non-coding repeat sequences [17,53]. Despite morphological and genomic differences, phylogenomic and phyletic analysis branch these viruses among other NCLDVs [119].

New members of the putative “*Pithoviridae*” family were isolated from Algerian, French, and Brazilian samples and named *Cedratviruses* [22,120,121]. *Cedratviruses* have elipsoid particles of ~1.0 µm but present some differences compared to other pithoviruses. Indeed, these viruses have two striate corks, one at each extremity of the particles [22] (Figure 5B). *Cedratviruses* replicate in *Acanthamoeba* cells, entering through phagocytosis and establishing a viral factory in the host cytoplasm, wherein a complex process of morphogenesis occurs, generating a viral progeny that is released mostly by cell lysis, although exocytosis is also possible [122]. Similar to pithoviruses, the cedratviruses have a large particle containing a relatively small circular genome (from 460,038 bp for *Brazilian Cedratvirus* to 589,068 for *Cedratvirus* A11), and are possible exceptions to the allometric law involving virion volume and genome length [121]. Those exceptions raised questions about DNA compaction and about macromolecules contained inside the particle particularly, where a lower density than the *Mimivirus* particle could be detected in *Pithovirus* [118]. Meanwhile, *Pithoviruses* and *Cedratviruses* reveal an extreme variation rate of their particles. Given the genomic variations we observed between *Cedratvirus* lineages, this particle size could be a powerful adaptation mechanism in light of evolution where the volume of the capsid does not become a constraint for the selection process and for genomic evolution. There are currently two lineages of *Cedratviruses*, a possible new genus of the expanding “*Pithoviridae*” family [121].

In 2018, *Orpheovirus* was isolated from a rat stool sample in *V. vermiformis* [21]. The *Orpheovirus* particle exhibited an ovoid shape with a size exceeding 1 µm (Figure 5C). It enclosed a circular genome (more than 1,47 Mb) larger than the ones of *Pithovirus* and *Cedratvirus*, despite a relative phylognetic proximity. Moreover, this genome revealed translational components and an expansion of gene contents compared to *Pithovirus*, *Cedratvirus*, *Marseilleviridae*, and *Irido-Ascoviridae* members [123].

More recently, various viruses and virophages were detected from metagenomic studies in this emerging *Pithoviridae* family. One of them was misannotated as a *Rickettsia* bacterium (*Misannotatedvirus*) [124]; 15 giant viruses were detected in Loki’s castle with a variation of size from 282,320 bp to 638,759 bp, and also detection of two virophages [125] and two others, *Solivirus* (276 kb) and *Solumvirus* (316 kb) from soil samples [52]. For these viruses and virophages, there are no structural data currently available and these must be determined by future isolates. With them, particles variations would be explained and may serve to measure gigantism impact on particle variation and genome evolution in this emerging *Pithoviridae* familly [123].

## 8. *Pandoraviruses*

The first two strains of pandoraviruses, *Pandoravirus salinus* and *Pandoravirus dulcis*, were described in 2013, isolated from samples collected on the coast of Chile, and from a freshwater pond near Melbourne, Australia [54]. Pandoraviruses are very different from other previously described giant viruses of amoebas, both by their morphological and genomic features. They have micrometer-sized ovoid-shaped particles, encompassing genomes ranging from 1.9 to 2.5 megabases [16,54,126,127]. Consequently, to the description of these two strains, *Pandoravirus inopinatum*, described few years earlier as an endosymbiont, was recognized as another strain of pandoravirus [128,129]. Three new strains of pandoraviruses, named *Pandoravirus massiliensis* BZ81c, *Pandoravirus pampulha* 8.8, and *Pandoravirus braziliensis* SL2, were isolated from soil samples collected from Pampulha lagoon and Belo Horizonte city, and from a soda lake (Soda Lake 2), Brazil [16]. Then, the group of pandoraviruses expanded rapidly with the isolation of *Pandoravirus quercus*, *Pandoravirus neocaledonia*, and *Pandoravirus macleodensis*, collected from ground soil in Marseille, France; from water of a mangrove in New Caledonia; and from a freshwater pond near Melbourne, Australia, respectively [130]. All the pandoravirus strains were isolated by co-culture on *Acanthamoeba castellanii*.

Furthermore, metagenomic studies showed that pandoraviruses are ubiquitous, as sequences related to these viruses were detected in environmental metagenomes collected worldwide, and even from human plasma [131,132,133,134,135,136,137].

*Pandoravirus* virion morphology stands apart from other giant viruses, by their ovoid particles of 1.5–2 µm in length with an apical pore via which the content of particles is emptied in the amoebal cytoplasm. The study of the replication cycle showed that pandoraviruses can recruit mitochondria and membranes, inducing a modification of the amoebal cytoplasm, especially into and around the viral factories. Interestingly, the authors also showed that pandoravirus infection induces a complete degradation of the host nucleus, after which viral factories start forming [138].

Pandoraviruses harbor a linear double-stranded DNA genome, with a high GC content ranging from 59 to 64% [16,126]. They encode a huge number of genes, from 1414 (*P. massiliensis*) to 2693 (*P. braziliensis*) open reading frames (ORFs), and some tRNAs [54]. A remarkable property is their high number of ORFans, reaching up to 84% for *P. salinus*. Among the other predicted proteins, more than half encode MORN, F Box, and Ankyrin repeats. The proportion of duplicated genes is very high, varying from 16% to more than half of the gene content (55%), according to the parameters used [16,130].

Genomes of pandoraviruses do not encompass any gene encoding for a known capsid, another interesting and unique characteristic distinguishing further away giant viruses from canonical viruses [54]. As determined by electron microscopy, the particles harbor a three-layered tegument-like envelope. *P. salinus* genome also encompasses a high number of transposable elements, named MITE for miniature inverted repeat transposable elements [139]. Two pangenome analyses were performed. The first included the genomes of *P. salinus*, *P. dulcis*, *P. inopinatum*, *P. massiliensis*, *P. braziliensis*, and *P. pampulha*, and revealed a very small core genome consisting of 4.7% of the total pangenome [16]. The second included *P. salinus*, *P. dulcis*, *P. inopinatum*, *P. quercus*, *P. neocaledonia*, and *P. macleodensis* [130]. It revealed 54–88% pairwise similarity between the different pandoravirus isolates, with 80% of orthologous genes being collinear. Thus, the group of pandoraviruses harbors a big open pangenome. It mainly consists of ORFans and hypothetical proteins, for some of which transcriptomics and proteomics experiments showed that they were transcribed and translated in proteins.

Transcriptomics experiments of *P. massiliensis* showed that at least 25% of the predicted genes are transcribed in the conditions used in the experiment. Two-thirds of the total reads provided by the sequencing were found 6 h post infection [16]. The transcripts included some ORFans and hypothetical proteins, i.e., predicted proteins for which no function is assigned. Proteomics studies revealed 424 viral gene products for *P. salinus*, 357 for *P. quercus*, 387 for *P. dulcis*, and 337 for *P. neocaledonia* [130]. In addition, it was reported that 25% of the genetic content of *P. massiliensis* was detected by transcriptomics, and 11.4% of viral gene products were found by proteomic analysis in virions, more than half of which belong to the core genome [16]. On the four strains of pandoraviruses simultaneously analyzed, the core proteome was estimated at 53% of the total protein clusters globally identified in all pandoravirions, whereas the core genome is only composed of 42% of the overall number of pandoravirus-encoded protein clusters [130].

These findings show that further analyses and investigations will be necessary to improve our knowledge on these very intriguing giant viruses.

## 9. Mobilome: From Isolation and Characterization of Mobile Genetic Elements to the Discovery of a System of Defense in Giant Viruses

In many features, giant viruses have similar characteristics to the three major branches of the tree of life. The mobilome is no exception and, in our laboratory, genetic elements were highlighted as independent or integrated genomic sequences.

### 9.1. The Virophage Discovery

**Figure 6:**
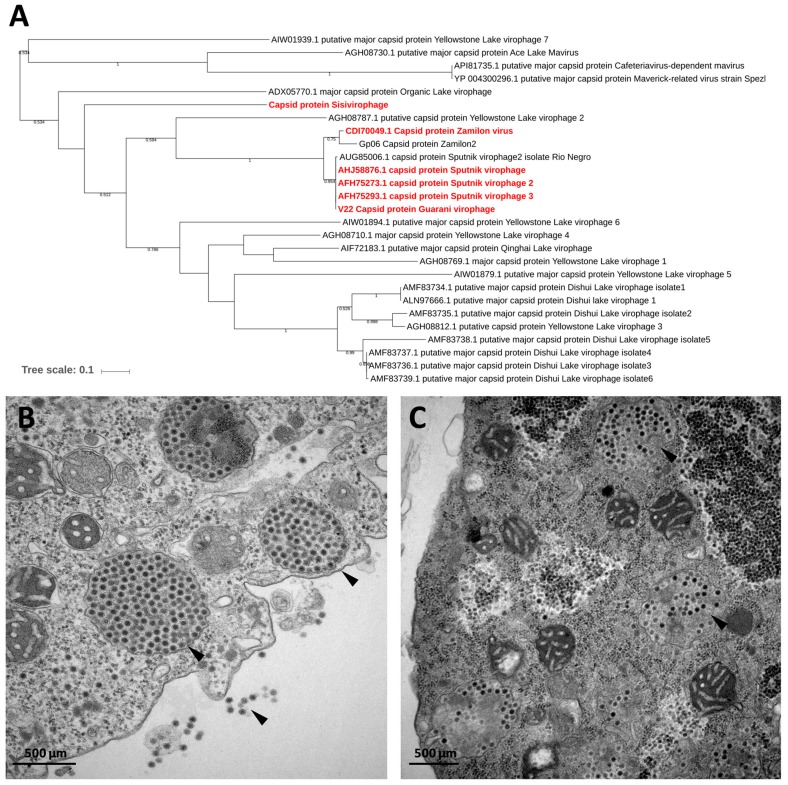
(**A**) Phylogenetic reconstruction based on the major capsid proteins of virophages. The virophages isolated in IHU are highlighted in red. The analysis was performed using MEGA version 7.0, applying the maximum-likelihood method and WAG model of evolution with 500 bootstrap replicates (cutoff ≥ 50). (**B**–**C**) Transmission electronic microscopy of virophage particles replicating in *Acanthamoeba castellanii* co-infected with a giant virus host (arrows). (B) Sputnik; (C) Zamilon.

The concept of a virophage as a parasitic agent that depends on and predates the replicative cycle of a host mimivirus dates back to 2008, when La Scola et al. observed the presence of small virions of approximately 50 nm, infiltrating the viral factory of *Acanthamoeba castellanii mamavirus*, a mimivirus belonging to the lineage A of the *Mimiviridae* family cultivated from waters of cooling towers in Paris, France [34]. This small viral entity was named Sputnik (Figure 6B). Sputnik founded a new class of microbial agents that are typical viruses characterized by their distinct reproduction strategy, known as virophages due to their functional analogy with bacteriophages [140,141]. Sputnik was produced from the same viral factory of the giant *Mamavirus* at a specific location and earlier stage of the co-infection cycle. Sputnik reproduction impaired the infectivity of its virus host, leading to a decrease in amoebae lysis. In addition, the morphogenesis of the mimivirus was significantly impacted resulting in a high abnormal particle production [34]. The replication of virophages relies on the presence of replication machinery of their viral host; therefore, they cannot replicate themselves in their host cell. Consequently, an isolation strategy which is based on the use of a cultivable helper giant virus was developed by our group. Our aim was to screen diverse clinical and environmental samples for the presence of virophages, to isolate them and analyze their structural, genomic, and biological features [25]. So far, nine virophage strains were successfully isolated from different origins including France, United States of America (USA), Brazil, Tunisia, and Germany using distinct sample sources including water, soil, contact lens rinse fluid, and plane tree [25,34,35,36,61,142,143]. Six of them were isolated by our team including three Sputnik strains [25,34,35], Zamilon [36], Guarani [37], and Sissivirophage (Unpublished under characterization). On the other hand, 57 uncultivated virophage population genomes were completely or partially assembled from diverse environmental metagenomes [73,144,145,146,147,148,149].

### 9.2. The Defense System MIMIVIRE

According to the Red Queen hypothesis [150], microorganisms are in a perpetual race to defend themselves against foreign genetic elements, and an unremitting arms race is engaged between the different hosts and invading entities. This evolutionary battle occurred between virophages and giant viruses. Based on this theory, we searched for sequences in the *Mimivirus* genome that could have been cannibalized and that would confer a selective advantage in the defense of *Mimivirus* against virophages [38]. As described above, several lineages (A, B, and C) were described in *Mimiviridae* and, among them, lineage A had the unique ability to resist against infection by a specific virophage, Zamilon [36]. According to our computational studies, we detected integrated and short repeated sequences of Zamilon in a gene (*R349*) present in the lineage A of *Mimivirus*. In addition, we localized, in the vicinity of these repeated sequences, genes that we assigned as a putative helicase and putative endonuclease (*R350* and *R354*). Although this system is distinct from the structure described in the well-known prokaryotic CRISPR/Cas system, a functional analogy was proposed, and we defined this system as a defense system in giant viruses, named MIMIVIRE (*Mimivirus* virophage resistance element) [38]. Based on these in silico predictions, experimental assays were subsequently conducted to validate our hypothesis. By targeting the three genes of the MIMIVIRE system, RNA silencing experiments confirmed their role and that of the virophage genomic insertion sequence as key elements of the MIMIVIRE system. Recently, transformation of a lineage A *Mimivirus* was successfully achieved and the deletion by knockout of the *R349* gene containing the repeated sequences of Zamilon restored the susceptibility of the infection by Zamilon [31]. These results confirmed our silencing experiments and definitely confirmed the *R349* gene as an essential element of MIMIVIRE. An alternative hypothesis based on protein-based interaction was also proposed to explain the resistance of *Mimivirus* against Zamilon [151]. Finally, recent experimental results described the structure of the nuclease R354. Structural analyses and mechanistic studies confirmed the critical role of this protein as a functional Cas4-like protein as initially proposed [152].

## 10. Giant Virus Genes in Metagenomes and Other Microorganisms

### 10.1. Giant Virus-Like Sequences in Eukaryotic Genomes

Genes from giant viruses were identified in the genomes of eukaryotic organisms, including amoebae and plants [153,154,155,156]. For instance, giant virus genes encoding major capsid proteins of giant viruses were detected in the genome sequences of various *Acanthamoeba* species, and other giant virus genes encoding family B DNA polymerase, DNA-dependent RNA polymerase subunits, or D5 helicase-primase in the genome of *Phycomitrella patens* and *Selaginella moellendorffii*. In the case of genomes of *Acanthamoeba* spp., results suggested that the sequence flow between these amoebae and giant viruses could be in both directions [156]. Moreover, using sequences encoding DNA-dependent RNA polymerase subunits as baits in BLAST searches, we found that sequences previously identified as *Hydra magnipapillata* or *Phytophtora parasitica* corresponded to giant viral sequences integrated in these eukaryotes’ DNA, or alternatively were possibly previously overlooked [157].

### 10.2. Giant Virus-Like Sequences in Metagenomes

The onset of metagenomics for the study of microorganims and its expansion coincided with the *Mimivirus* discovery and the extension of giant virus diversity [158]. The power of metagenomics was tremendously enhanced by next-generation sequencing technologies. We detected, on several occasions, giant-virus-like sequences in metagenomes generated from various environmental, animal, and human samples. These results helped highlight that giant viruses are common in our biosphere. We were able to assemble ~40% of the genome of a Zamilon virophage from sequences generated from a bioreactor metagenome, in which sequences best matching with mimiviruses were concurrently detected [144]. We also implemented a tool, MG-Digger, to explore metagenomes and detect giant-virus-like sequences [131]. It homogenizes the format of metagenomes and then performs automated reciprocal BLAST hits, including comparison with BLAST searches conducted in the NCBI GenBank nucleotide or protein sequence databases. We notably tested MG-Digger on metagenomes generated from marine water and sewage, but also human blood samples. We detected sequences most related to various giant viruses including *Mimiviruses* and *Marseilleviruses*, but also more recently discovered representative members such as *Faustoviruses*, *Pithoviruses*, and *Pandoraviruses*, as well as virophage-like sequences. In addition, we detected sequences from putative giant virus relatives in the NCBI GenBank environmental protein sequence database (env nr), using giant virus sequences encoding a DNA-dependent RNA polymerase subunit, as well as reconstructed putative ancestral sequences for this protein as baits [157]. Metagenome analyses further provided evidence of the presence of giant viruses in humans. Thus, the fortuitous detection of *Marseillevirus*-like sequences in a metagenome from the stools of a young and healthy Senegalese man led to the isolation, for the first time, a giant virus named *Senegalvirus* from this human sample [42,159]. Then, metagenomics of blood samples from 10 blood donors from southern France led to detecting that 2.5% of the viral metagenome best matched with *Marseillevirus*-like sequences and could be assembled in two 10–13 kbp-large contigs closely related to the genome of the *Marseillevirus* prototype strain [43]. Thus, giant virus sequences, which contain large proportions of ORFans, contributed to enlighten the “dark matter” of environmental and human metagenomes since their discovery. This recently prompted other teams to assert that mimiviruses may be more abundant than prokaryotic organisms in marine waters [13] and could represent substantial fractions of the DNA virome from human specimens [160,161].

## 11. Giant Viruses in Humans

The presence of giant viruses in humans was documented especially for *Mimiviruses* and *Marseilleviruses*, the two families of giant viruses of amoebas first described.

### 11.1. Marseilleviruses

The first giant virus isolated from a human sample was *Senegalvirus*, a close relative of *Marseillevirus*, from a stool of a healthy young Senegalese man [42]. It was serendipitously found during a metagenomic and culturomic study. Then, sequences closed to *Marseillevirus* were detected in the metagenome generated from the blood of blood donors from France [43]. In the same study, *Marseillevirus* was detected by PCR in 10% of the 20 blood samples tested. However, these results were controversial, with other studies showing negative results for all blood samples tested by PCR, with the same primers used [162]. Seroprevalence studies showed that the exposition of humans to *Marseilleviruses* was unexpectedly high with rates varying between 1.7 and 15% [43,44,104], and reaching up to 23% in multitransfused individuals [44]. These data suggested then that the contacts of *Marseilleviruses* with humans are frequent.

*Marseilleviruses* were also detected in lymphoid organs in three reports. A *Marseillevirus*-like organism was detected in a lymph node of an 11-month-old boy who presented an adenitis of undetermined etiology [45], in the lymph node from a 30-year-old woman with Hodgkin lymphoma, and in the tonsils of a 20-year-old patient with neurologic disorders [46,47]. In addition, it was described, in this latter case, the first evidence of a prolonged carriage of *Marseillevirus* in humans. These reports may raise questions about the pathogenicity of these *Marseilleviruses*, particularly in the case of lymphomas. The prolonged carriage, the presence in the blood, the detection in pathologic lymph nodes are arguments for a possible causal link between the virus and the disease. Many other infectious agents, as the Epstein–Barr virus, are linked with lymphomas, and the question on the link between *Marseillevirus* and lymphomas could be open; however, further investigations are required. Furthermore, a murine model confirmed the prolonged detection of the virus in a mammalian organism, after an experimental inoculation by pulmonary or intravenous routes [105].

Moreover, *Marseillevirus* sequences were also detected in metagenomic studies in buccal mucosa, retro-auricular crease, vagina, and stools from healthy individuals [159,160].

### 11.2. Mimiviruses

*Mimiviruses* were first isolated in an amoebal co-culture of water samples from cooling towers during the investigation of an outbreak of pneumonia [11]. Then, the association between *Mimiviruses* and pneumonia in humans was explored. Firstly, seroprevalence studies showed that the rates found in community-acquired pneumonia and nosocomial pneumonia were 9.7% and 10%, respectively, versus 2.3% and 3% in control patients [87,163]. Moreover, seroconversions were observed in up to 8% and 22% of nosocomial pneumonia and community-acquired pneumonia patients, while any seroconversion was observed in control patients. In addition, a study also showed that *Mimivirus* could be the fourth most common etiology of acquired nosocomial and community pneumonia [164].

The seroconversion of a laboratory technician who handled huge quantities of *Acanthamoeba polyphaga mimivirus* and developed pneumonia, for which all other pathogens were negative, was a strong argument for the association between *Mimivirus* and pneumonia [165]. Indeed, an experimental murine model confirmed these data through the development of pneumonia in mice inoculated by the giant virus [166]. Moreover, two strains of mimiviruses were isolated from two pneumonia patients and three mimiviruses were detected by PCR in three cases of pneumonia [39,40,167]. Recently, a mimivirus was also isolated in the urines of a kidney-transplant recipient [41]. However, these associations between giant viruses and a disease were controversial, as other studies failed to detect mimivirus by PCR in all nasopharyngeal aspirates and bronchoalveolar liquid samples [88,168]. The rarity of these findings may be due to the high genetic variability of the genomes of mimiviruses [169]. Furthermore, it was demonstrated that *Mimivirus* was able to inhibit the regulation of IFN-stimulated genes, thus evading the IFN system. These results suggest that *Mimivirus* and humans have host–pathogen interactions [170]. Another team showed that 22% of rheumatoid arthritis patients had antibodies against mimivirus collagen versus 6% in control patients, showing that *Mimivirus* exposure is a risk factor for the development of auto immunity against collagen [171].

Recent metagenomic studies also reported the presence of *Mimivirus* sequences in human stools, nasopharyngeal aspirates from patients with respiratory tract infections, buccal mucosa, saliva and retroauricular crease, vagina, blood from healthy people, and even in blood from patients with liver diseases [159,160]. Rampelli et al. also found that *Mimivirus* sequences were the most represented viral sequences together with *Poxviridae* sequences in the human gut.

## 12. Conclusion

This 10th-year anniversary special issue of the journal *Viruses* gave us the opportunity to overview 15 years of research and discoveries on giant viruses. The most recent metagenomic works [51,52] or the renewed interpretation of old observations [172] suggest that these early years were only the beginning of discoveries in a vast field of life that probably holds great surprises in the years to come.

## Figures and Tables

**Table 1:** Input of the University Hospital Institute (IHU) Mediterranee Infection to the knowledge of giant viruses. (* First described by the laboratory; UPC: unpublished under characterization).

	Description Year		References
**Giant virus isolation**	*Acanthamoeba polyphaga mimivirus* (Lineage A)*	2003	La Scola et al. [11]
	*Moumouvirus* (Lineage B)*	2010	La Scola et al. [14]
	CE11 *Mimivirus* (Lineage C, Megavirus)*	2010	La Scola et al. [14]
	Marseillevirus*	2009	Boyer et al. [15]
	*Pandoravirus*	2018	Aherfi et al. [16]
	*Pithovirus*	2016	Levasseur et al. [17]
	Faustovirus*	2015	Reteno et al. [18]
	Yasminevirus*		Bajrai (SFP)
	Tupanvirus*	2018	Abrahão et al. [19]
	Kaumoebavirus*	2016	Bajrai et al. [20]
	Orpheovirus*	2018	Andreani et al. [21]
	Cedratvirus*	2016	Andreani et al. [22]
	Pacmanvirus*	2017	Andreani et al. [23]
	*Clandestinovirus*		(UPC)
	Fadolivirus*		(UPC)
	Sissivirus*		(UPC)
	Usurpativirus*		(UPC)
**Technique**	High-throughput isolation on agar		Boughalmi et al. [24]
	Use of a reporter giant virus to isolate virophage		Gaia et al. [25]
	High-throughput isolation using flow cytometry		Bou Kahlil et al. [26]
	Mixture separation using fluorescence-activated cell sorting (FACS)		Bou Khalil et al. [27]
**Other original features**	Drastic reduction of *Mimivirus* genome under allopatric conditions		Boyer et al. [28]
	*Mimivirus* translation initiation factor 4a modify viral protein translation		Bekliz et al. [29]
	Giant virus with unexpected translation apparatus		Abrahão et al. [19] Abrahão et al. [19] Sobhy et al. [30]
	Giant virus leading to ribosomal shutdown of host and not host protozoa		
	First silencing in giant virus		Mougari et al. [31]
	First knock-out of a giant virus		Boughalmi et al. [32]
	Isolation of giant virus from insects		Temmam et al. [33]
**Giant virus mobilome**	First isolation of virophage		La Scola et al. [34]
	Isolation and description of 5 other virophages		Desnues et al. [35], Gaia et al. [36],
			Mougari et al. [37]
	First description of the provirophage and transpoviron elements		Desnues et al. [35]
	First description of a defense system of giant viruses against virophages		Levasseur et al. [38]
**Giant viruses and human pathology**	Isolation of *Mimivirus* in broncho-alveoloar lavage and stool samples of patients suffering pneumonia		Saadi et al. [39]
			Saadi et al. [40]
	Isolation of *Mimivirus* in a urine sample from a patient with kidney transplantation		Moal et al. [41]
	Isolation of *Marseillevirus* in a stool sample of a patient from Senegal		Lagier et al. [42]
	Detection of a virus closely related to *Marseillevirus* using viral metagenomics, in blood from blood donors, and evidence of serological prevalence in blood transfusions		Popgeorgiev et al. [43,44]
	Highlighting with immunohistochemistry of a *Marseillevirus*-like organism in a lymph node from a child with unexplained adenitis		Popgeorgiev et al. [45]
	Association between *Marseillevirus* infection and lymphoma occurrence		Aherfi et al. [46]
	Association between *Marseillevirus* and neurologic disorder		Aherfi et al. [47]

**Table 2:** Evolution of the co-culture strategies over the years: tools, host panels, and sample diversity.

Improvements of the Co-Culture Strategies				
Tools			Cell Hosts	Samples
Technique	Detection and Identification	Co-Culture Supports		
2003: Amoebal enrichment method	ECP detection under an inverted microscope Hemacolor, DAPI Molecular biology Electron microscopy	Shell vials	*Acanthamoeba polyphaga*	Water samples
2013: High-throughput isolation on agar	ECP detection by the naked eye Hemacolor, DAPI Molecular biology Electron microscopy	Agar plate	*Acanthamoeba polyphaga*	Soil and water samples
2016: High-throughput isolation using flow cytometry and mixture sorting	Flow cytometry: ECP detection Presumptive identification Sorting of viral mixtures Electron microscopy	Microplates (96 wells)	*Acanthamoeba castellanii* *Vermamoeba vermiformis* *Acanthamoeba polyphaga* *Acanthamoeba griffini* *Acanthamoeba quina* *Acanthamoeba mauritanensis* *Acanthamoeba divionensis* *Acanthamoeba culbertsoni* *Cafeteria roenbergensis* *Dictyostelium discoideum* *Tetrahymena hyperangularis* *Poterioochromonas malhamensis*	Diverse ecosystems: sea water, lake water, rain water, soil, sewage, human samples, algae, animal stool, fungi, plants, insects, permafrosts, etc.

**Table 3:** List of the 15 published *Marseillevirus* isolates.

Lineage	Virus Name	Source	Country/ Continent	Description Year
Lineage A	*Marseillevirus*	[15]	France/Europa	2009
	*Fontaine Saint Charles virus*	[14]	France/Europa	
	*Senegalvirus*	[42]	Senegal/Africa	2012
	*Canne8 virus*	[91]	France/Europa	2013
	*Melbourne virus*	[92]	Australia/Oceania	2014
	*Tokyovirus*	[93]	Japan/Asia	2016
Lineage B	*Lausannevirus*	[94]	France/Europa	2011
	*Noumeavirus*	[95]	New Caledonia/Oceania	2017
	*Port-Miou virus*	[96]	France/Europa	2015
	*Kurlavirus*	[97]	India/Asia	2017
Lineage C	*Tunisvirus*	[24]	Tunisia/Africa	2014
	*Insectomime virus*	[32]	Tunisia/Africa	2013
Lineage D	*Brazilian marseillevirus*	[99]	Brazil/America	2016
Lineage E	*Golden marseillevirus*	[100]	Brazil/America	2016
Unclassified	*Marseillevirus Shangai isolate 1*	GenBank MG827395	China/Asia	2018
